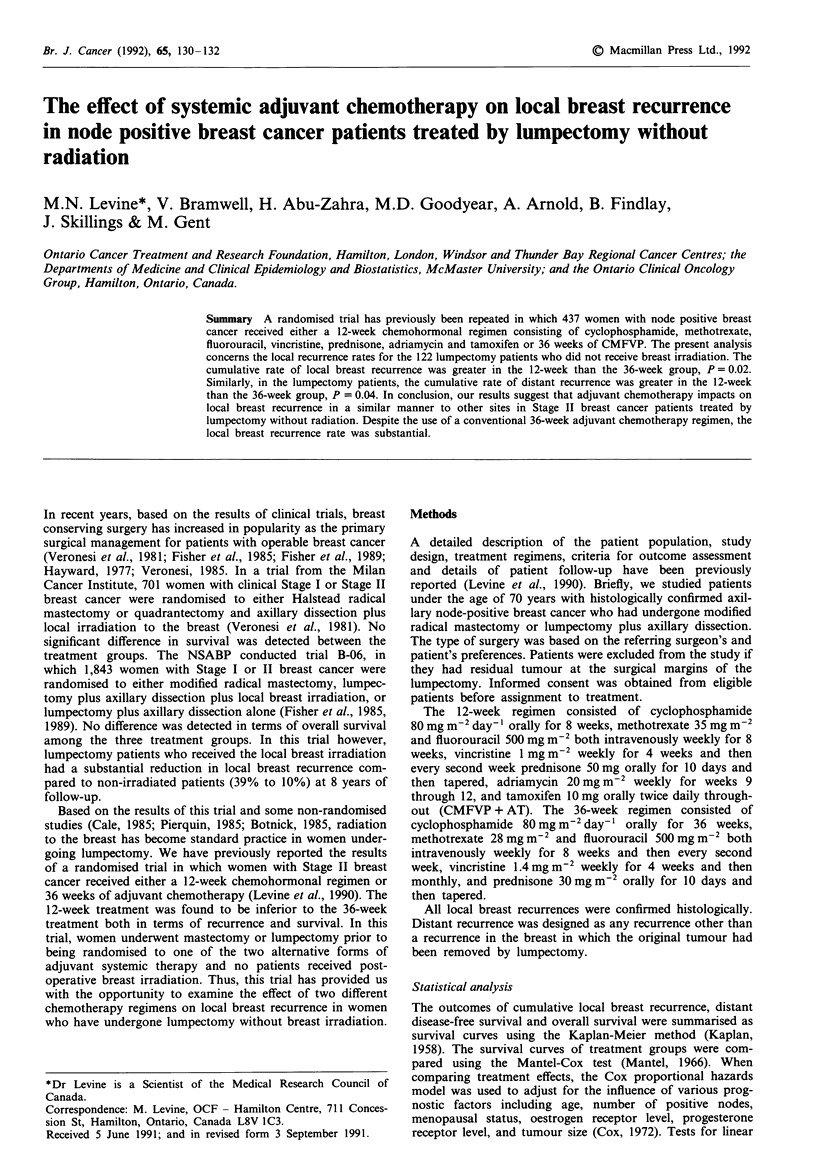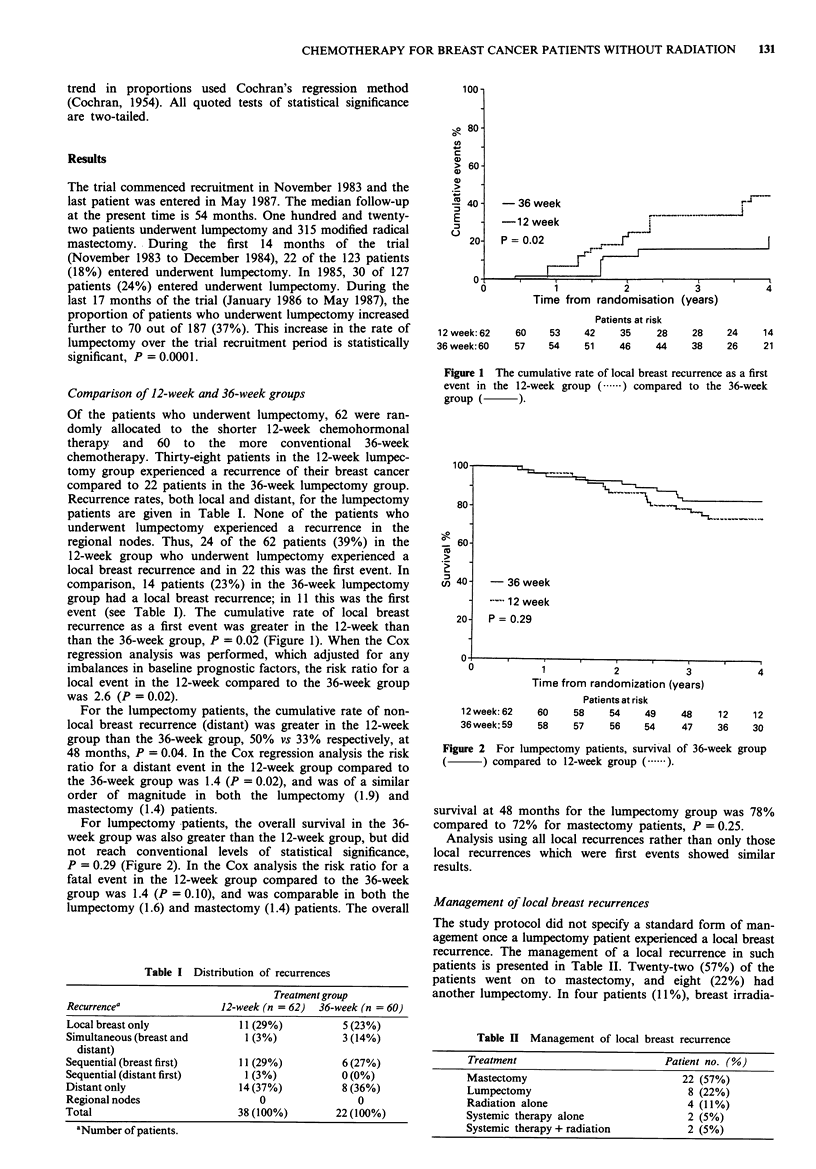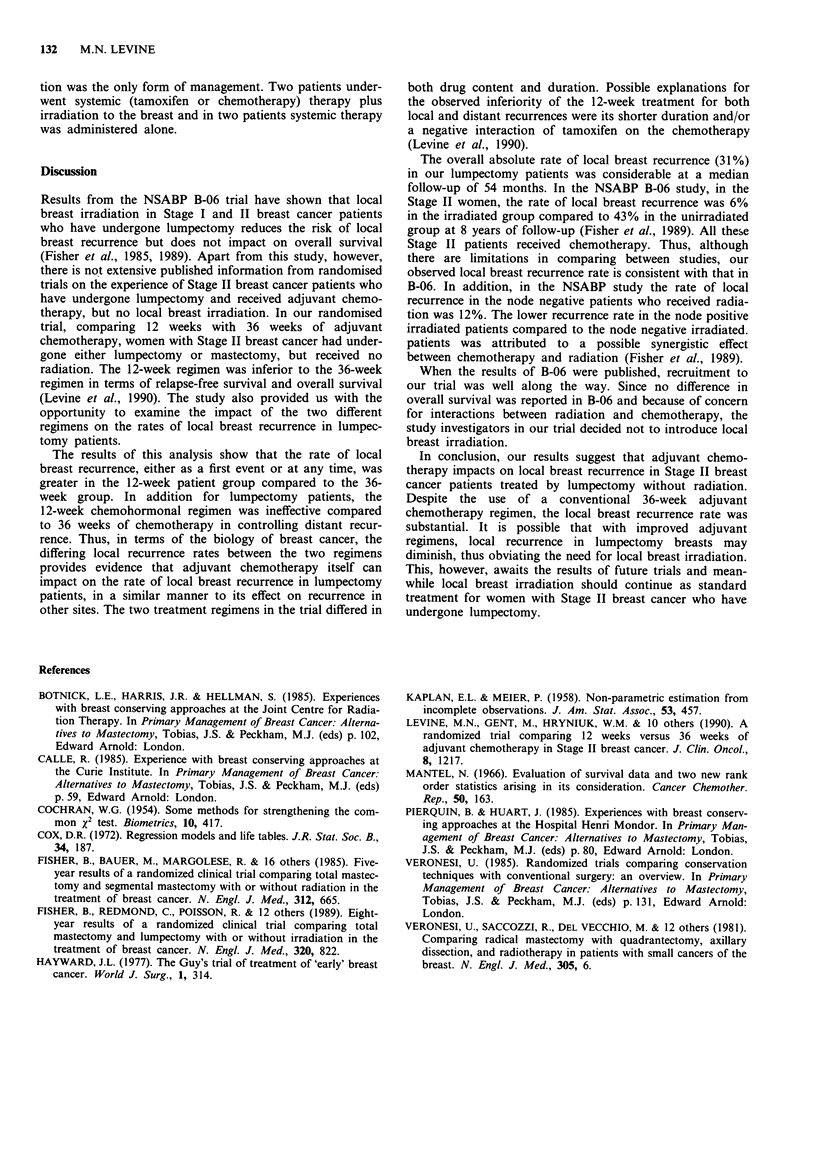# The effect of systemic adjuvant chemotherapy on local breast recurrence in node positive breast cancer patients treated by lumpectomy without radiation.

**DOI:** 10.1038/bjc.1992.25

**Published:** 1992-01

**Authors:** M. N. Levine, V. Bramwell, H. Abu-Zahra, M. D. Goodyear, A. Arnold, B. Findlay, J. Skillings, M. Gent

**Affiliations:** Ontario Cancer Treatment and Research Foundation, Hamilton, London, Canada.

## Abstract

A randomised trial has previously been repeated in which 437 women with node positive breast cancer received either a 12-week chemohormonal regimen consisting of cyclophosphamide, methotrexate, fluorouracil, vincristine, prednisone, adriamycin and tamoxifen or 36 weeks of CMFVP. The present analysis concerns the local recurrence rates for the 122 lumpectomy patients who did not receive breast irradiation. The cumulative rate of local breast recurrence was greater in the 12-week than the 36-week group, P = 0.02. Similarly, in the lumpectomy patients, the cumulative rate of distant recurrence was greater in the 12-week than the 36-week group, P = 0.04. In conclusion, our results suggest that adjuvant chemotherapy impacts on local breast recurrence in a similar manner to other sites in Stage II breast cancer patients treated by lumpectomy without radiation. Despite the use of a conventional 36-week adjuvant chemotherapy regimen, the local breast recurrence rate was substantial.


*Br. J. Cancer* (1992), **65**, 130–132 © Macmillan Press Ltd., 1992

### The effect of systemic adjuvant chemotherapy on local breast recurrence in node positive breast cancer patients treated by lumpectomy without radiation

M.N. Levine*, V. Bramwell, H. Abu-Zahra, M.D. Goodyear, A. Arnold, B. Findlay, J. Skillings & M. Gent

*Ontario Cancer Treatment and Research Foundation, Hamilton, London, Windsor and Thunder Bay Regional Cancer Centres; the Departments of Medicine and Clinical Epidemiology and Biostatistics, McMaster University; and the Ontario Clinical Oncology Group, Hamilton, Ontario, Canada.*

**Summary** A randomised trial has previously been repeated in which 437 women with node positive breast cancer received either a 12-week chemohormonal regimen consisting of cyclophosphamide, methotrexate, fluorouracil, vincristine, prednisone, adriamycin and tamoxifen or 36 weeks of CMFVP. The present analysis concerns the local recurrence rates for the 122 lumpectomy patients who did not receive breast irradiation. The cumulative rate of local breast recurrence was greater in the 12-week than the 36-week group, $P = 0.02$. Similarly, in the lumpectomy patients, the cumulative rate of distant recurrence was greater in the 12-week than the 36-week group, $P = 0.04$. In conclusion, our results suggest that adjuvant chemotherapy impacts on local breast recurrence in a similar manner to other sites in Stage II breast cancer patients treated by lumpectomy without radiation. Despite the use of a conventional 36-week adjuvant chemotherapy regimen, the local breast recurrence rate was substantial.

In recent years, based on the results of clinical trials, breast conserving surgery has increased in popularity as the primary surgical management for patients with operable breast cancer (Veronesi *et al.*, 1981; Fisher *et al.*, 1985; Fisher *et al.*, 1989; Hayward, 1977; Veronesi, 1985. In a trial from the Milan Cancer Institute, 701 women with clinical Stage I or Stage II breast cancer were randomised to either Halstead radical mastectomy or quadrantectomy and axillary dissection plus local irradiation to the breast (Veronesi *et al.*, 1981). No significant difference in survival was detected between the treatment groups. The NSABP conducted trial B-06, in which 1,843 women with Stage I or II breast cancer were randomised to either modified radical mastectomy, lumpectomy plus axillary dissection plus local breast irradiation, or lumpectomy plus axillary dissection alone (Fisher *et al.*, 1985, 1989). No difference was detected in terms of overall survival among the three treatment groups. In this trial however, lumpectomy patients who received the local breast irradiation had a substantial reduction in local breast recurrence compared to non-irradiated patients (39% to 10%) at 8 years of follow-up.

Based on the results of this trial and some non-randomised studies (Cale, 1985; Pierquin, 1985; Botnick, 1985, radiation to the breast has become standard practice in women undergoing lumpectomy. We have previously reported the results of a randomised trial in which women with Stage II breast cancer received either a 12-week chemohormonal regimen or 36 weeks of adjuvant chemotherapy (Levine *et al.*, 1990). The 12-week treatment was found to be inferior to the 36-week treatment both in terms of recurrence and survival. In this trial, women underwent mastectomy or lumpectomy prior to being randomised to one of the two alternative forms of adjuvant systemic therapy and no patients received post-operative breast irradiation. Thus, this trial has provided us with the opportunity to examine the effect of two different chemotherapy regimens on local breast recurrence in women who have undergone lumpectomy without breast irradiation.

*Dr Levine is a Scientist of the Medical Research Council of Canada.

Correspondence: M. Levine, OCF – Hamilton Centre, 711 Concession St, Hamilton, Ontario, Canada L8V 1C3.

Received 5 June 1991; and in revised form 3 September 1991.

### Methods

A detailed description of the patient population, study design, treatment regimens, criteria for outcome assessment and details of patient follow-up have been previously reported (Levine *et al.*, 1990). Briefly, we studied patients under the age of 70 years with histologically confirmed axillary node-positive breast cancer who had undergone modified radical mastectomy or lumpectomy plus axillary dissection. The type of surgery was based on the referring surgeon's and patient's preferences. Patients were excluded from the study if they had residual tumour at the surgical margins of the lumpectomy. Informed consent was obtained from eligible patients before assignment to treatment.

The 12-week regimen consisted of cyclophosphamide $80\ \mathrm{mg\ m^{-2}\ day^{-1}}$ orally for 8 weeks, methotrexate $35\ \mathrm{mg\ m^{-2}}$ and fluorouracil $500\ \mathrm{mg\ m^{-2}}$ both intravenously weekly for 8 weeks, vincristine $1\ \mathrm{mg\ m^{-2}}$ weekly for 4 weeks and then every second week prednisone 50 mg orally for 10 days and then tapered, adriamycin $20\ \mathrm{mg\ m^{-2}}$ weekly for weeks 9 through 12, and tamoxifen 10 mg orally twice daily throughout (CMFVP + AT). The 36-week regimen consisted of cyclophosphamide $80\ \mathrm{mg\ m^{-2}\ day^{-1}}$ orally for 36 weeks, methotrexate $28\ \mathrm{mg\ m^{-2}}$ and fluorouracil $500\ \mathrm{mg\ m^{-2}}$ both intravenously weekly for 8 weeks and then every second week, vincristine $1.4\ \mathrm{mg\ m^{-2}}$ weekly for 4 weeks and then monthly, and prednisone $30\ \mathrm{mg\ m^{-2}}$ orally for 10 days and then tapered.

All local breast recurrences were confirmed histologically. Distant recurrence was designed as any recurrence other than a recurrence in the breast in which the original tumour had been removed by lumpectomy.

### *Statistical analysis*

The outcomes of cumulative local breast recurrence, distant disease-free survival and overall survival were summarised as survival curves using the Kaplan-Meier method (Kaplan, 1958). The survival curves of treatment groups were compared using the Mantel-Cox test (Mantel, 1966). When comparing treatment effects, the Cox proportional hazards model was used to adjust for the influence of various prognostic factors including age, number of positive nodes, menopausal status, oestrogen receptor level, progesterone receptor level, and tumour size (Cox, 1972). Tests for linear trend in proportions used Cochran's regression method (Cochran, 1954). All quoted tests of statistical significance are two-tailed.

### Results

The trial commenced recruitment in November 1983 and the last patient was entered in May 1987. The median follow-up at the present time is 54 months. One hundred and twenty-two patients underwent lumpectomy and 315 modified radical mastectomy. During the first 14 months of the trial (November 1983 to December 1984), 22 of the 123 patients (18%) entered underwent lumpectomy. In 1985, 30 of 127 patients (24%) entered underwent lumpectomy. During the last 17 months of the trial (January 1986 to May 1987), the proportion of patients who underwent lumpectomy increased further to 70 out of 187 (37%). This increase in the rate of lumpectomy over the trial recruitment period is statistically significant, $P = 0.0001$.

### *Comparison of 12-week and 36-week groups*

Of the patients who underwent lumpectomy, 62 were randomly allocated to the shorter 12-week chemohormonal therapy and 60 to the more conventional 36-week chemotherapy. Thirty-eight patients in the 12-week lumpectomy group experienced a recurrence of their breast cancer compared to 22 patients in the 36-week lumpectomy group. Recurrence rates, both local and distant, for the lumpectomy patients are given in Table I. None of the patients who underwent lumpectomy experienced a recurrence in the regional nodes. Thus, 24 of the 62 patients (39%) in the 12-week group who underwent lumpectomy experienced a local breast recurrence and in 22 this was the first event. In comparison, 14 patients (23%) in the 36-week lumpectomy group had a local breast recurrence; in 11 this was the first event (see Table I). The cumulative rate of local breast recurrence as a first event was greater in the 12-week than than the 36-week group, $P = 0.02$ (Figure 1). When the Cox regression analysis was performed, which adjusted for any imbalances in baseline prognostic factors, the risk ratio for a local event in the 12-week compared to the 36-week group was 2.6 ($P = 0.02$).

For the lumpectomy patients, the cumulative rate of non-local breast recurrence (distant) was greater in the 12-week group than the 36-week group, 50% *vs* 33% respectively, at 48 months, $P = 0.04$. In the Cox regression analysis the risk ratio for a distant event in the 12-week group compared to the 36-week group was 1.4 ($P = 0.02$), and was of a similar order of magnitude in both the lumpectomy (1.9) and mastectomy (1.4) patients.

For lumpectomy patients, the overall survival in the 36-week group was also greater than the 12-week group, but did not reach conventional levels of statistical significance, $P = 0.29$ (Figure 2). In the Cox analysis the risk ratio for a fatal event in the 12-week group compared to the 36-week group was 1.4 ($P = 0.10$), and was comparable in both the lumpectomy (1.6) and mastectomy (1.4) patients. The overall survival at 48 months for the lumpectomy group was 78% compared to 72% for mastectomy patients, $P = 0.25$.

Analysis using all local recurrences rather than only those local recurrences which were first events showed similar results.

**Table I** Distribution of recurrences

| Recurrence[a] | Treatment group 12-week ($n = 62$) | 36-week ($n = 60$) |
|---|---|---|
| Local breast only | 11 (29%) | 5 (23%) |
| Simultaneous (breast and distant) | 1 (3%) | 3 (14%) |
| Sequential (breast first) | 11 (29%) | 6 (27%) |
| Sequential (distant first) | 1 (3%) | 0 (0%) |
| Distant only | 14 (37%) | 8 (36%) |
| Regional nodes | 0 | 0 |
| Total | 38 (100%) | 22 (100%) |

[a]Number of patients.

**Figure 1** The cumulative rate of local breast recurrence as a first event in the 12-week group (······) compared to the 36-week group (———).

**Figure 2** For lumpectomy patients, survival of 36-week group (———) compared to 12-week group (······).

### *Management of local breast recurrences*

The study protocol did not specify a standard form of management once a lumpectomy patient experienced a local breast recurrence. The management of a local recurrence in such patients is presented in Table II. Twenty-two (57%) of the patients went on to mastectomy, and eight (22%) had another lumpectomy. In four patients (11%), breast irradia-

**Table II** Management of local breast recurrence

| Treatment | Patient no. (%) |
|---|---|
| Mastectomy | 22 (57%) |
| Lumpectomy | 8 (22%) |
| Radiation alone | 4 (11%) |
| Systemic therapy alone | 2 (5%) |
| Systemic therapy + radiation | 2 (5%) |

tion was the only form of management. Two patients underwent systemic (tamoxifen or chemotherapy) therapy plus irradiation to the breast and in two patients systemic therapy was administered alone.

### Discussion

Results from the NSABP B-06 trial have shown that local breast irradiation in Stage I and II breast cancer patients who have undergone lumpectomy reduces the risk of local breast recurrence but does not impact on overall survival (Fisher *et al.*, 1985, 1989). Apart from this study, however, there is not extensive published information from randomised trials on the experience of Stage II breast cancer patients who have undergone lumpectomy and received adjuvant chemotherapy, but no local breast irradiation. In our randomised trial, comparing 12 weeks with 36 weeks of adjuvant chemotherapy, women with Stage II breast cancer had undergone either lumpectomy or mastectomy, but received no radiation. The 12-week regimen was inferior to the 36-week regimen in terms of relapse-free survival and overall survival (Levine *et al.*, 1990). The study also provided us with the opportunity to examine the impact of the two different regimens on the rates of local breast recurrence in lumpectomy patients.

The results of this analysis show that the rate of local breast recurrence, either as a first event or at any time, was greater in the 12-week patient group compared to the 36-week group. In addition for lumpectomy patients, the 12-week chemohormonal regimen was ineffective compared to 36 weeks of chemotherapy in controlling distant recurrence. Thus, in terms of the biology of breast cancer, the differing local recurrence rates between the two regimens provides evidence that adjuvant chemotherapy itself can impact on the rate of local breast recurrence in lumpectomy patients, in a similar manner to its effect on recurrence in other sites. The two treatment regimens in the trial differed in both drug content and duration. Possible explanations for the observed inferiority of the 12-week treatment for both local and distant recurrences were its shorter duration and/or a negative interaction of tamoxifen on the chemotherapy (Levine *et al.*, 1990).

The overall absolute rate of local breast recurrence (31%) in our lumpectomy patients was considerable at a median follow-up of 54 months. In the NSABP B-06 study, in the Stage II women, the rate of local breast recurrence was 6% in the irradiated group compared to 43% in the unirradiated group at 8 years of follow-up (Fisher *et al.*, 1989). All these Stage II patients received chemotherapy. Thus, although there are limitations in comparing between studies, our observed local breast recurrence rate is consistent with that in B-06. In addition, in the NSABP study the rate of local recurrence in the node negative patients who received radiation was 12%. The lower recurrence rate in the node positive irradiated patients compared to the node negative irradiated. patients was attributed to a possible synergistic effect between chemotherapy and radiation (Fisher *et al.*, 1989).

When the results of B-06 were published, recruitment to our trial was well along the way. Since no difference in overall survival was reported in B-06 and because of concern for interactions between radiation and chemotherapy, the study investigators in our trial decided not to introduce local breast irradiation.

In conclusion, our results suggest that adjuvant chemotherapy impacts on local breast recurrence in Stage II breast cancer patients treated by lumpectomy without radiation. Despite the use of a conventional 36-week adjuvant chemotherapy regimen, the local breast recurrence rate was substantial. It is possible that with improved adjuvant regimens, local recurrence in lumpectomy breasts may diminish, thus obviating the need for local breast irradiation. This, however, awaits the results of future trials and meanwhile local breast irradiation should continue as standard treatment for women with Stage II breast cancer who have undergone lumpectomy.

### References

BOTNICK, L.E., HARRIS, J.R. & HELLMAN, S. (1985). Experiences with breast conserving approaches at the Joint Centre for Radiation Therapy. In *Primary Management of Breast Cancer: Alternatives to Mastectomy*, Tobias, J.S. & Peckham, M.J. (eds) p. 102, Edward Arnold: London.

CALLE, R. (1985). Experience with breast conserving approaches at the Curie Institute. In *Primary Management of Breast Cancer: Alternatives to Mastectomy*, Tobias, J.S. & Peckham, M.J. (eds) p. 59, Edward Arnold: London.

COCHRAN, W.G. (1954). Some methods for strengthening the common $\chi^2$ test. *Biometrics*, **10**, 417.

COX, D.R. (1972). Regression models and life tables. *J.R. Stat. Soc. B.*, **34**, 187.

FISHER, B., BAUER, M., MARGOLESE, R. & 16 others (1985). Five-year results of a randomized clinical trial comparing total mastectomy and segmental mastectomy with or without radiation in the treatment of breast cancer. *N. Engl. J. Med.*, **312**, 665.

FISHER, B., REDMOND, C., POISSON, R. & 12 others (1989). Eight-year results of a randomized clinical trial comparing total mastectomy and lumpectomy with or without irradiation in the treatment of breast cancer. *N. Engl. J. Med.*, **320**, 822.

HAYWARD, J.L. (1977). The Guy's trial of treatment of 'early' breast cancer. *World J. Surg.*, **1**, 314.

KAPLAN, E.L. & MEIER, P. (1958). Non-parametric estimation from incomplete observations. *J. Am. Stat. Assoc.*, **53**, 457.

LEVINE, M.N., GENT, M., HRYNIUK, W.M. & 10 others (1990). A randomized trial comparing 12 weeks versus 36 weeks of adjuvant chemotherapy in Stage II breast cancer. *J. Clin. Oncol.*, **8**, 1217.

MANTEL, N. (1966). Evaluation of survival data and two new rank order statistics arising in its consideration. *Cancer Chemother. Rep.*, **50**, 163.

PIERQUIN, B. & HUART, J. (1985). Experiences with breast conserving approaches at the Hospital Henri Mondor. In *Primary Management of Breast Cancer: Alternatives to Mastectomy*, Tobias, J.S. & Peckham, M.J. (eds) p. 80, Edward Arnold: London.

VERONESI, U. (1985). Randomized trials comparing conservation techniques with conventional surgery: an overview. In *Primary Management of Breast Cancer: Alternatives to Mastectomy*, Tobias, J.S. & Peckham, M.J. (eds) p. 131, Edward Arnold: London.

VERONESI, U., SACCOZZI, R., DEL VECCHIO, M. & 12 others (1981). Comparing radical mastectomy with quadrantectomy, axillary dissection, and radiotherapy in patients with small cancers of the breast. *N. Engl. J. Med.*, **305**, 6.